# Comprehensive Genomic Identification and Expression Analysis of the Phosphate Transporter (PHT) Gene Family in Apple

**DOI:** 10.3389/fpls.2017.00426

**Published:** 2017-03-30

**Authors:** Tingting Sun, Mingjun Li, Yun Shao, Lingyan Yu, Fengwang Ma

**Affiliations:** State Key Laboratory of Crop Stress Biology for Arid Areas, College of Horticulture, Northwest A&F UniversityYangling, China

**Keywords:** apple, phosphate transporters, gene family, phosphorus stress, drought, expression

## Abstract

Elemental phosphorus (Pi) is essential to plant growth and development. The family of phosphate transporters (PHTs) mediates the uptake and translocation of Pi inside the plants. Members include five sub-cellular phosphate transporters that play different roles in Pi uptake and transport. We searched the Genome Database for Rosaceae and identified five clusters of phosphate transporters in apple (*Malus domestica*), including 37 putative genes. The MdPHT1 family contains 14 genes while MdPHT2 has two, MdPHT3 has seven, MdPHT4 has 11, and MdPHT5 has three. Our overview of this gene family focused on structure, chromosomal distribution and localization, phylogenies, and motifs. These genes displayed differential expression patterns in various tissues. For example, expression was high for *MdPHT1;12, MdPHT3;6*, and *MdPHT3;7* in the roots, and was also increased in response to low-phosphorus conditions. In contrast, *MdPHT4;1, MdPHT4;4*, and *MdPHT4;10* were expressed only in the leaves while transcript levels of *MdPHT1;4, MdPHT1;12*, and *MdPHT5;3* were highest in flowers. In general, these 37 genes were regulated significantly in either roots or leaves in response to the imposition of phosphorus and/or drought stress. The results suggest that members of the PHT family function in plant adaptations to adverse growing environments. Our study will lay a foundation for better understanding the PHT family evolution and exploring genes of interest for genetic improvement in apple.

## Introduction

Phosphorus (Pi) is a key nutrient for plant growth and development (Raghothama, 1999) and is also an essential component of fertilizers used to sustain modern agriculture. In plants, Pi is part of important biomolecules and, in the form of phosphate, pyrophosphate, adenosine triphosphate, adenosine diphosphate, or adenosine monophosphate, has a crucial role in energy transfer and metabolic regulation. Globally, many soils are deficient in Pi and levels of available-Pi seldom exceed 10 μM even in fertile soils (Bieleski, 1973). Meanwhile, the Pi concentration in plant cell cytoplasm is generally greater than 10 mM (Raghothama, 1999). Therefore, plants must have specialized transporters to move Pi from the soil into cells against a large concentration gradient at the root–soil interface. Genome sequence analyses and experimental evidence have indicated that plants contain numerous Pi transporter families, including PHT1, PHT2, PHT3, PHT4, and PHT5, which are distinguished by their protein sequences, structures, locations, and functions (Schachtman et al., 1998; Mimura, 1999; Rausch and Bucher, 2002; Knappe et al., 2003; Guo et al., 2008; Liu et al., 2011, 2016; Zhang et al., 2016).

The PHT1 family has been the most widely studied in plants, and PHT1 proteins represent a group of phosphate carriers usually found in the plasma membrane (Pao et al., 1998). The first high-H^+^/Pi phosphate transporter identified in higher plants was AtPT1 from *Arabidopsis thaliana* (Muchhal et al., 1996). This gene has significant roles in the uptake of phosphorus from the soil (Lopez-Arredondo et al., 2014). Analyses of expressed sequence tags (ESTs) and genome sequences have revealed nine genes in *Arabidopsis* that share similarity with *AtPT1*, among which *PHT1;1* transcripts is the most abundant (Mudge et al., 2002). Its overexpression increases Pi uptake in *Arabidopsis* (Wang et al., 2014). *Arabidopsis thaliana PHT2;1* was the first member of the PHT2 family to be identified. AtPHT2;1 is a chloroplast phosphate transporter (Ferro et al., 2002; Versaw and Harrison, 2002) and also a low-affinity Pi transporter (Daram et al., 1999). Its activity affects Pi allocations and translocation within the plant and modulates the expression of Pi-starvation response genes (Versaw and Harrison, 2002). It also is a positive control for light-induced expression (Rausch et al., 2004). The third family of plant Pi transporters is localized to the mitochondria and includes the highly conserved PHT3 (Laloi, 1999). Three *PHT3* genes have been identified in *Arabidopsis* (Rausch and Bucher, 2002). Within the PHT4 family, six members have been described from *Arabidopsis* (Guo et al., 2008). These genes are expressed in both roots and leaves. In addition to AtPHT4;1, a candidate thylakoid membrane-localized transporter, other transporters may be found in that organelle (Miyaji et al., 2015). PHT4;2 contributes to Pi transport in isolated root plastids, and starch accumulations are reduced in the roots and leaves of mutant plants (Irigoyen et al., 2011). AtPHT4;4 is a chloroplast-localized ascorbate transporter (Miyaji et al., 2015) and is induced by light exposure (Wang et al., 2011). AtPHT4;6 transports Pi out of the Golgi lumenal space to be recycled after release from glycosylation (Cubero et al., 2009). Allocation of phosphate, as mediated by PHT4;6, is critical for preventing the onset of dark-induced senescence (Hassler et al., 2016; Sebastian et al., 2016). Three *Arabidopsis* SYG1, PHO81, and XPR1 (SPX)-Major Facility Superfamily (MFS) proteins residing in the tonoplast are thought to form the phosphate transporter 5 family (Liu et al., 2016). Plants that over-express *PHT5s* show diminished growth and greater accumulations of Pi in their vacuoles relative to the cytosol, indicating transient misregulation of Pi-starvation response genes (Liu et al., 2016). In particular, AtPHT5;1 plays a prominent role in Pi accumulation. Similar to those in *Arabidopsis*, SPX-MFS proteins in rice (*Oryza sativa*) are localized in the tonoplast (Wang et al., 2015). There, PHT5s in the vacuolar membranes regulate cytoplasmic Pi homeostasis and are required for fitness and plant growth.

Apple (*Malus domestica*) is the fourth most economically significant woody fruit plant after *Citrus* sp., *Vitis vinifera*, and *Musa* L. (Hummer and Janick, 2009). Phosphate is an important nutrient for apple crops because it helps drive flowering, as well as fruit set, quality, and yield. Because many soils around the world are phosphate-deficient, abundant phosphatic fertilizers are applied to fields each year (Goldstein, 1992). In production areas within China, drought is the most challenging stress for apple trees (Hayano-Kanashiro et al., 2009). Therefore, it is urgent that researchers develop plants with enhanced efficiency of soil phosphorus use under such conditions. In doing so, one can also begin to reduce the environmental pollution caused by over-fertilization. One main approach to these problems is to improve the capacity of apple roots to absorb phosphorus. Because Pi is moved from the soil into plant cells in response to excess phosphate, genomic analyses have been conducted with Pi transporter families in *Arabidopsis* and rice. However, little is known about that gene family in woody plant species such as apple, which has a larger genome when compared with *Arabidopsis* and rice. Online publication of that genome (Velasco et al., 2010) has provided new tools for accelerating the identification of genes and other functional elements in apple (Troggio et al., 2012). Here, we isolated 37 *MdPHT* genes in *Malus*. Our goals were to examine gene structures, predict chromosome localizations, study phylogeny, and monitor patterns of transcriptional expression in roots, stem, leaves, shoot tips, flowers, young fruits, and mature fruits and in response to various abiotic stresses. The objective was to present a foundation for further functional dissection of MdPHTs so that genetic engineering approaches can been applied in efforts to improve the efficiency of phosphate uptake by stressed apple plants.

## Materials and methods

### Database searches for the identification and chromosome locations of PHT family members in apple

Previously reported phosphate transporter proteins for *Arabidopsis thaliana, Oryza sativa*, and *Populus trichocarpa* were used as queries against the apple genome database (http://genomics.research.iasma.it/). After overlapping sequences were removed, the genome annotations of *Malus domestica* were downloaded from that database. The protein sequences were aligned by ClustalX (ftp://ftp-igbmc.u-strasbg.fr/pub/ClustalX/) with default parameters, and were submitted to the Conserved Domain Database (http://www.ncbi.nlm.nih.gov/Structure/cdd/wrpsb.cgi), Pfam (http://pfam.sanger.ac.uk/), and SMART (http://smart.emblheidelberg.de/).

To verify our findings, we screened the apple EST database at the National Center for Biotechnology Information (NCBI) database (http://www.ncbi.nlm.nih.gov/guide/). Coding sequences identified from those genomic data were inspected and revised according to information from a database of *M. domestica* ESTs in the NCBI webserver. To identify protein sequences, we used the tools of the ExPASY Molecular Biology Server (http://web.expasy.org/protparam/) and TMHMM Server v. 2.0 (http://www.cbs.dtu.dk/services/TMHMM-2.0/). Locational data for PHT genes were retrieved from the Genome Database for Rosaceae (http://www.rosaceae.org/).

### Multiple sequence alignments, phylogenetic analysis, exon/intron organization, and localization of MdPHT proteins

Multiple alignments were performed with default parameters in the DNAMAN program (Lynnon Biosoft, USA). Full-length protein sequences from *Arabidopsis*, rice, and *P*. *trichocarpa* were downloaded from the NCBI protein database to study their evolutionary relationships. Phylogenetic trees were drawn with the MEGA6 program (Testerink and Munnik, 2011), using the Neighbor-Joining (NJ) method (Saitou and Nei, 1987) with Poisson corrections and 1,000 replications for the bootstrap analysis. A map of exon/intron organization was obtained from the online Gene Structure Display Server (http://gsds.cbi.pku.edu.cn/), applying the method for coding sequences and genomic sequences. Localization of each PHT was predicted via WoLF PSORT (http://www.genscript.com/wolf-psort.html).

### Plant materials and treatments

*Malus hupehensis* var. *pingyiensis* is tolerant of waterlogged conditions. Because of its strong tendency for apomixes, few differences occur among seedlings (Li, 2001). Therefore, we used 6-year-old plants, the spacing of apple trees were 2 × 4 m, grown in an orchard at the Horticulture Experimental Station of Northwest A&F University, Yangling, Shaanxi, China, to study tissue-specific expression. Before the experiments began, all trees were well watered weekly and were supplied with compound fertilizer. Healthy and uniform plants were selected for sampling. Samples were taken of new roots, young stem segments, mature leaves from the middle portion of shoots, flowers (5 days after blooming, DBA), shoot tips, young fruits (15 DAB) and mature fruits (122 DAB). Roots, stems, mature leaves and young fruits were sampled at 15 DAB. Flowers were taken from apple trees 5 DAB. All samples were harvested from the south side of the tree canopy between 10:00 a. m. and 11:00 a. m., under full sun exposure.

For the hydroponics experiments, seeds were stratified in sand at 0 to 4°C for 60 d. After germinating, they were planted in individual plastic pots (12 × 12 cm) filled with sand and then placed in a greenhouse for 60 d under natural lighting and temperature conditions. To ensure that the seedling responses were consistent when exposed to our phosphorus and/or drought treatments, we planted three times as many seeds as were needed for these trials. The pots were moved out to an experimental field for 60 d under natural lighting and temperature conditions. Beginning at the second-true-leaf stage, the seedlings were irrigated every 4 d with a 1/2-strength Hoagland nutrient solution (Hoagland and Arnon, 1950). After 60 days outdoors, selected seedlings of similar size (with 6–8 leaves) were transferred to black plastic basins (52 × 37 × 15 cm), each containing 13 L of a 1/2-strength Hoagland nutrient solution. The basins were placed in a greenhouse under natural light and at day/night temperatures of 23 to 25°C/15 to 18°C. The nutrient solution was aerated each hour with an air pump and the dissolved oxygen concentration was maintained at 8.0 to 8.5 mg L^−1^. The pH of the nutrient solution was adjusted to 6.0 ± 0.1 by adding diluted H_2_SO_4_, and the solution was refreshed every 4 d. After 10 d of such pre-cultivation, stress treatments were initiated. Seedlings of *Malus hupehensis* var. *pingyiensis* were randomly assigned to six groups (*n* = 54 plants per treatment) each stress treatment had three biological replicates, every replicates contains 18 plants: (1) Control, standard 1/2-strength Hoagland nutrient solution supplemented with 500 μM KH_2_PO_4_; (2) Low-P treatment, 1/2-strength Hoagland nutrient solution plus 5 μM KH_2_PO_4_; (3) High-P treatment, 1/2-strength Hoagland nutrient solution with 5 mM KH_2_PO_4_; (4) Mild drought stress, generated by adding PEG 6000 to the 1/2-strength Hoagland solution and adjusting the osmotic potential to −0.75 MPa; (5) Combination of low-P and drought stress, 1/2-strength Hoagland nutrient solution (5 μM KH_2_PO_4_) and the osmotic potential adjusted to −0.75 MPa; or (6) Combination of and high-P and drought stress, 1/2-strength Hoagland nutrient solution (5 mM KH_2_PO_4_) and the osmotic potential adjusted to −0.75 MPa. On Day 15 of the experimental period, roots and leaves were harvested respectively.

### RNA extraction and cDNA synthesis

All tissue samples were quickly frozen in liquid nitrogen and stored at −80°C. Total RNA was extracted according to the cetyl trimethyl ammonium bromide method (Chang et al., 1993). Residual DNA was removed by treating with RNase-free DNase I (Invitrogen, USA). The concentration of RNA was accurately quantified after this DNase I treatment, and 5 μg of total RNA was separated via 1.2% agarose gel electrophoresis to assess its quality and integrity. The RNA concentration and purity were measured with a spectrophotometer (NANODROP 2000c; ThermoScientific, USA). First-strand cDNAs were synthesized with 1 μg of total RNA, using a SYBR Prime Script RT-PCR Kit II (TaKaRa, Japan). After reverse-transcription, the reaction product was diluted 10-fold with sterile water as backup.

### Molecular cloning of PHT genes in apple

Leaf cDNA was used as template for amplifying the *MdPHT* sequences. Specific primers (Table S1) for gene cloning were designed based on the revised putative sequences. Polymerase chain reactions (PCRs) were performed with PrimeSTAR® HS DNA Polymerase (TaKaRa), and amplification conditions were empirically optimized. The PCR products were added to the 3′-termini using TaqDNA Polymerase (Fermentas, USA) and cloned into the pMD19-T vector (TaKaRa). Afterward, positive clones were sequenced. Gene nomenclature was assigned based on chromosome orders.

### Expression analysis of apple PHT genes

To obtain expression profiles for *MdPHT*s in different tissues and in response to phosphorus and/or drought treatment, we performed quantitative real-time PCR (qRT-PCR) on an iQ5.0 instrument (Bio-Rad, USA), using SYBR Green qPCR kits (TaKaRa) according to the manufacturer's instructions. The *Actin* gene served as our standard. Gene-specific primers were designed for these amplifications (Table S1). All reactions included 10.0 μL of SYBR® Premix Ex Taq™ (TaKaRa), 1.0 μL of cDNA template, 0.4 μL of each specific primer, and 8.2 μL of ddH_2_O_2_, made up to a 20-μL volume. The PCR conditions involved an initial 95°C for 3 min; then 40 cycles of 95°C for 20 s, 56°C for 20 s, and 72°C for 20 s. Based on three separate RNA extracts from three biological replications samples, each qRT-PCR was conducted three times to minimize inherent errors. Means and standard deviations were calculated from the results of three biological replicates. The relative expression levels of all *MdPHT* genes were calculated by the 2^−ΔΔCT^ method (Livak and Schmittgen, 2001).

## Results

### Identification of MdPHT genes and chromosomal distribution of family members in apple

To confirm the existence of *PHT* genes in apple, we employed previously identified protein sequences for *Arabidopsis* and rice in a broad search against apple genomics databases. In all, 43 candidates were found in apple. These included MDP0000926667 and MDP0000197466 on chr1, MDP0000278249 and MDP0000315320 on chr5, MDP0000209897 and MDP0000156379 on chr6, MDP0000613331 and MDP0000190109 on chr7, MDP0000255882 and MDP0000794044 on chr8, and MDP0000214285 and MDP0000315710 on chr17. Because each of those six pairs was close together on their respective chromosomes and the protein sequences of each pairing were highly similar, we speculated that they were tandem-duplicated genes. Therefore, each pair was treated as a single complete PHT sequence. This resulted in 37 *PHT* genes being identified in apple. The MdPHT1 family accounted for 14 genes, MdPHT2 for two, MdPHT3 for seven, MdPHT4 for 11, and MdPHT5 for five. Our BLAST analysis against the Pfam database showed that all proteins belong to the MFS family. Table 1 presents each gene name, locus, genome position, length of the open reading frame (ORF), numbers of exons and amino acids, isoelectric point (PI), molecular weight (MW), and GenBank Accession Number.

**Table 1:** **Properties of PHTs identified from the *Malus domestica* genome**.

**Gene**	**Locus**	**Genome position**	**ORF length (bp)**	**Number of exons**	**Number of amino acids**	**PI**	**MW (kDa)**	**TMS**	**Subcellular location prediction**	**GenBank accession number**
MdPHT1;1	MDP0000926667	chr1:27634315.27636074	1,593	2	530	9.10	58.4100	12	plas: 7, E.R.: 3, chlo: 1, cyto: 1, vacu: 1	KX853027
	MDP0000197466	chr1:27661004.27662763						13		
MdPHT1;2	MDP0000717558	chr5:12316999.12318615	1,554	1	517	9.04	56.7735	13	plas: 12, chlo: 2	
MdPHT1;3	MDP0000278249	chr5:12319459.12321075	1,617	1	538	7.10	59.1949	12	plas: 8, golg: 2, cyto: 1, extr: 1, vacu: 1	
	MDP0000315320	chr5:12313596.12318317								
MdPHT1;4	MDP0000301845	chr6:711908.717794	1,548	2	515	8.40	57.1660	12	plas: 5, E.R.: 4, cyto: 3, chlo: 1	
MdPHT1;5	MDP0000166425	chr7:21568115.21569785	1,671	1	556	8.51	60.5061	12	plas: 7, cyto: 4, chlo: 1, vacu: 1	
MdPHT1;6	MDP0000935883	chr7:24791009.24792817	1,593	2	530	8.82	58.6313	12	cyto: 6, plas: 5, chlo: 2	
MdPHT1;7	MDP0000261121	chr7:3288949.3296707	1,665	2	554	8.64	60.4202	12	plas: 8, vacu: 2, golg: 2, cyto: 1	
MdPHT1;8	MDP0000508371	chr10:18616063.18617679	1,617	1	538	8.55	59.2277	13	plas: 8, vacu: 3, cyto: 1, E.R.: 1	
MdPHT1;9	MDP0000560198	chr10:3641243.3642856	1,614	1	537	8.80	58.8809	12	plas: 9, E.R.: 2, cyto: 1, vacu: 1	KX853028
MdPHT1;10	MDP0000298304	chr11:3674586.3676193	1,608	1	535	8.21	58.5742	12	plas: 8, vacu: 4, golg: 2	
MdPHT1;11	MDP0000137073	chr12:24306561.24308138	1,578	1	525	8.40	57.5193	12	plas: 6, vacu: 4, extr: 2, cyto: 1	
MdPHT1;12	MDP0000075043	chr13:25913713.25915275	1,566	1	521	8.95	57.2580	13	plas: 6, extr: 2, E.R.: 2, golg: 2, cyto: 1	
MdPHT1;13	MDP0000746621	chr16:18349145.18354640	1,566	1	521	8.94	57.1166	13	plas: 5, vacu: 3, extr: 2, golg: 2, cyto: 1	
MdPHT1;14	MDP0000570409	unanchored:69974310.69975926	1,617	1	538	7.10	59.1088	13	plas: 8, E.R.: 2, golg: 2, cyto: 1	
MdPHT2;1	MDP0000685160	chr13:6839259.6842290	1,773	3	590	9.29	62.3888	12	chlo: 8, mito: 5	KX853029
MdPHT2;2	MDP0000820780	chr16:4955018.4958051	1,770	3	589	9.28	62.0641	12	chlo: 7.5, chlo_mito: 7, mito: 5.5	KX853030
MdPHT3;1	MDP0000146063	chr6:16581740.16583921	1,137	1	378	9.34	40.1830	7	chlo: 12, mito: 2	KX853031
MdPHT3;2	MDP0000255882	chr8:9914829.9917203	930	1	309	9.25	34.0794	7	chlo: 10, plas: 2, cyto: 1	
	MDP0000794044	chr8:9914886.9917260								
MdPHT3;3	MDP0000943164	chr9:416293.418984	1,176	1	391	9.22	41.9267	8	chlo: 11, vacu: 1.5, E.R._vacu: 1.5	
MdPHT3;4	MDP0000288329	chr10:13756151.13760013	1,017	1	338	9.43	37.3726	7	cyto: 7, chlo: 5, nucl: 1	
MdPHT3;5	MDP0000158576	chr14:20375725.20378339	1,254	1	417	9.07	44.8298	7	chlo: 14	
MdPHT3;6	MDP0000214285	chr17:242374.244787	1,113	1	370	9.42	39.5592	7	chlo: 13	
	MDP0000315710	chr17:242568.244978						12		
MdPHT3;7	MDP0000137665	chr17:267601.270014	1,113	1	370	9.42	39.5590	12	chlo: 13	
MdPHT4;1	MDP0000166477	chr1:9348653.9353821	1,785	9	594	9.29	65.7600	12	plas: 9, E.R.: 3, vacu: 1	
MdPHT4;2	MDP0000238253	chr2:31053368.31054697	1,233	3	410	9.75	44.6647	12	plas: 9, E.R.: 3, chlo: 2	
MdPHT4;3	MDP0000283990	chr2:31055920.31057249	1,230	2	409	9.71	44.3734	12	plas: 6, chlo: 4, E.R.: 4	KX853032
MdPHT4;4	MDP0000145529	chr3:6532205.6535488	1569	12	522	8.85	57.6530	12	E.R.: 7, plas: 3.5, cyto_plas: 2.5, vacu: 2	KX853033
MdPHT4;5	MDP0000256650	chr4:14770520.14777413	1,806	9	601	9.13	65.6217	12	nucl: 6, chlo: 5.5, chlo_mito: 3.5, pero: 2	
MdPHT4;6	MDP0000181017	chr6:106890.110323	1,665	9	554	9.68	60.6479	12	plas: 6.5, E.R.: 6, cyto_plas: 4	KX853034
MdPHT4;7	MDP0000613331	chr7:2889614.2890942	1,329	1	442	9.68	47.8724	12	plas: 7, E.R.: 4, chlo: 3	KX853035
	MDP0000190109	chr7:2886236.2887132								
MdPHT4;8	MDP0000260623	chr11:7071134.7074883	1,497	10	498	9.31	54.6269	12	plas: 5, E.R.: 5, vacu: 2, chlo: 1	KX853036
MdPHT4;9	MDP0000238657	chr11:7285443.7292800	1,515	13	504	9.96	54.6579	12	E.R.: 6, plas: 5.5, cyto_plas: 3.5, mito: 1	
MdPHT4;10	MDP0000173837	chr15:36887273.36891911	1,827	10	608	9.63	67.3806	12	chlo: 6, plas: 5, E.R.: 2	
MdPHT4;11	MDP0000152286	chr15:8962214.8967426	1,596	15	531	6.51	57.6730	12	plas: 6, chlo: 5, E.R.: 2	
MdPHT5;1	MDP0000231565	chr2:20653881.20657726	2,100	10	699	8.14	77.7790	12	plas: 6, E.R.: 4, nucl: 1, cyto: 1, vacu: 1	KX853037
MdPHT5;2	MDP0000209897	chr6:13326795.13331281	2,013	9	670	6.58	75.0290	12	plas: 4, E.R.: 4, chlo: 3, nucl: 2	KX853038
	MDP0000156379	chr6:13328723.13333161								
MdPHT5;3	MDP0000199977	chr10:4037017.4041739	2,199	11	732	5.69	81.3195	12	plas: 8, E.R.: 4, cyto: 1	KX853039

**Figure 1:**
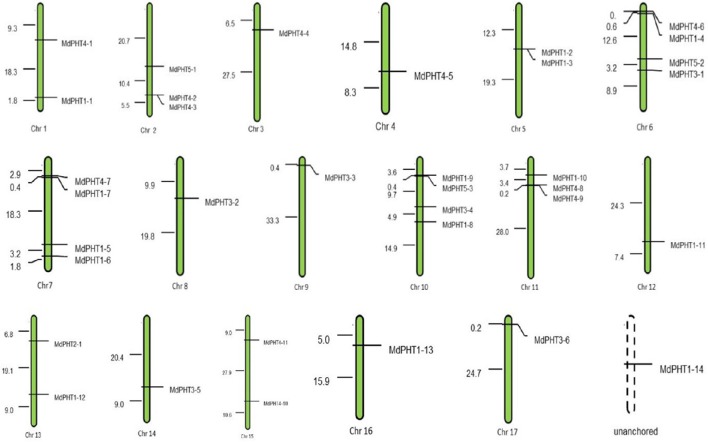
**Positions of PHT gene family members on apple chromosomes**.

In all, 36 *MdPHT* genes are distributed on 17 apple chromosomes while one gene is not anchored (Figure 1). Chromosomes 1, 5, 13, 15, 16, and 17 each contain two genes; chr 2 and 11 have three each; chr 3, 4, 8, 9, 12, and 14 have one each; and chr 6, 7, and 10 have four each (Figure 1).

**Figure 2:**
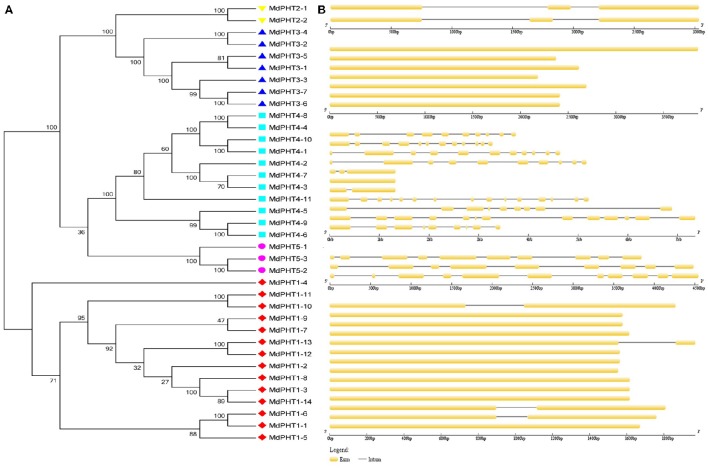
**(A)** Phylogenetic analysis. Different colors and shapes indicate genes within individual clusters. **(B)** Schematic diagram of exon/intron structures for PHT genes in apple. Diagram was made via Gene Structure Display Server, applying method for coding sequences and corresponding genome sequences. Yellow boxes and gray lines represent exons and introns, respectively.

The full-length cDNA sequences were compared with corresponding genomic DNA sequences to determine the numbers and positions of exons and introns within each *MdPHT* gene (Figures 2A,B). Of the 37 total coding sequences, 18 showed no intron while the other coding sequences were disrupted by one to 15 introns each.

Values for PI ranged from 5.69 to 9.96, while the 37 putative proteins had molecular weights of 34.0 to 81.3 kDa. These MdPHT protein sequences contained seven to 13 the transmembrane domain (TMDs). While MdPHT1 members were approximately 520 to 540 aa long, lengths for MdPHT2s were approximately 590, 300, to 400 for MdPHT3s (i.e., the shortest proteins), 400 to 590 for MdPHT4s, and 670 to 730 for MdPHT5s. A search of the Transport Classification Database (http://www.tcdb.org/analyze.php) identified seven to 13 transmembrane segments (TMSs) in the apple PHT family.

### Molecular cloning of MdPHT genes in apple

We cloned 13 of the 37 putative *MdPHT* genes and provided to NCBI (Table 1). Comparison of the sequences with published apple gene predictions revealed four anomalies, including redundant fragments and deletions. Such redundancies were detected in the middle portions of MDP0000146063, MDP0000283990, and MDP0000181017. Moreover, the predicted sequences of MDP0000145529 lacked a sequence region compare with the cloned gene from *M. hupehensis*. Nevertheless, these cloned sequences shared high similarity with the predicted coding sequences (CDS) in the apple genome, regardless of improperly predicted, redundant, or absent fragments.

### Multiple sequence alignments and phylogenetic analysis

**Figure 3:**
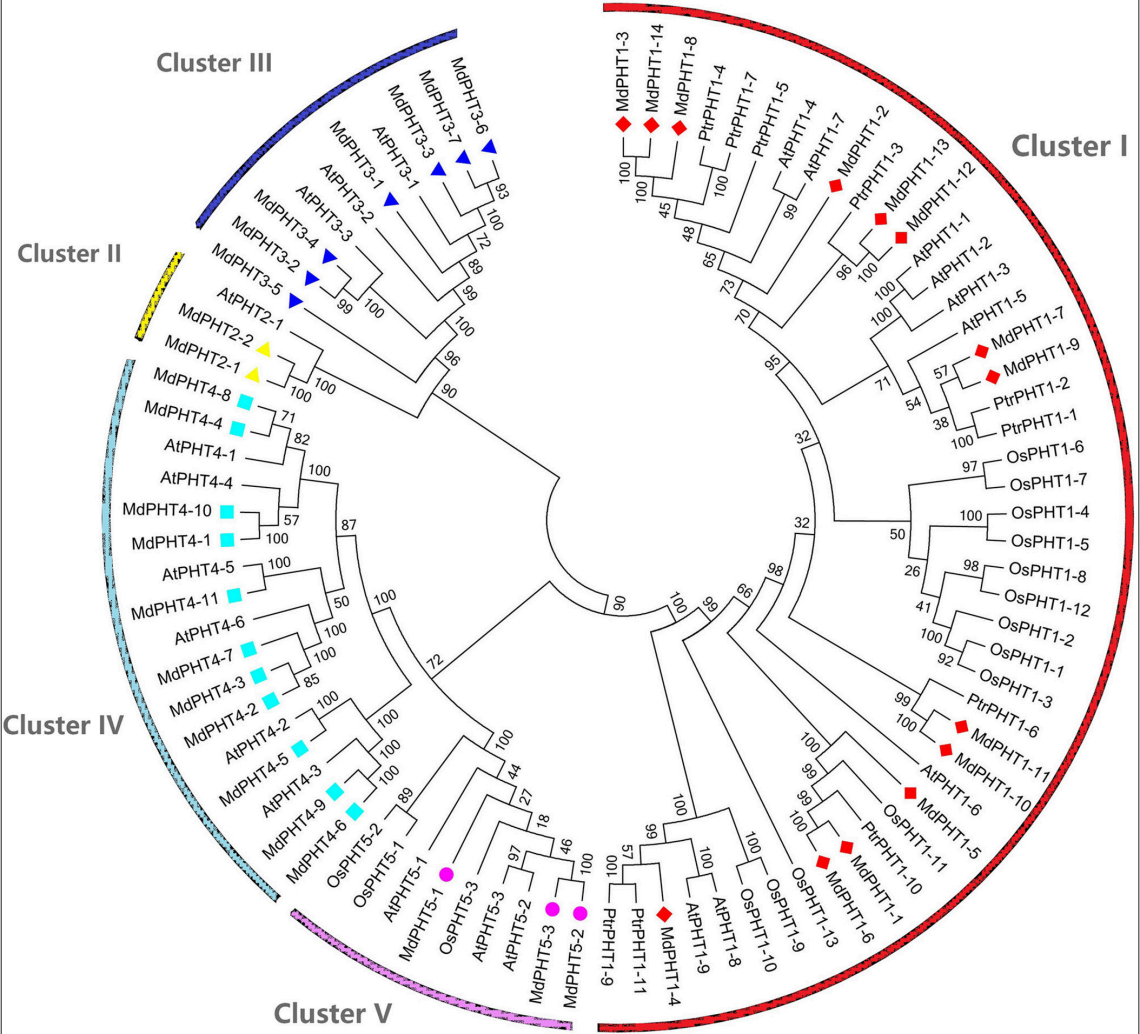
**Phylogenetic analyses of PHT genes in *Arabidopsis thaliana* (At), *Oryza sativa* (Os), *Malus domestica* (Md), and *Populus trichocarpa* (Ptr)**. Unrooted phylogenetic tree was constructed by NJ method, using MEGA6.0 program.

To investigate the evolutionary and phylogenetic relationships among PHTs in apple and other species at the molecular level, we compared their full-length protein sequences. Phylogenetic trees constructed by the NJ method (Figure 3) indicated that these PHT proteins could be divided into five clusters (I, II, III, IV, and V). Cluster I contained 14 members; Cluster II, two members; Cluster III, seven; Cluster IV, 11; and Cluster V, three. Those five clusters resembled the grouping of PHT families (PHT1 through PHT5) in *Arabidopsis* (Rausch and Bucher, 2002; Guo et al., 2008; Liu et al., 2016). This tree demonstrated that the *MdPHT*s are more closely related to dicot *Arabidopsis* and *P. trichocarpa* than to the monocot rice. We also confirmed that apple has 10 sets of paralogous genes: *MdPHT2;1/MdPHT2;2, MdPHT3;4/MdPHT3;2, MdPHT3;7/MdPHT3;6, MdPHT4;8/MdPHT4;4, MdPHT4;10/MdPHT4;1, MdPHT4;9/MdPHT4;6, MdPHT5;1/MdPHT5;2/MdPHT5;3, MdPHT1;11/MdPHT1;10, MdPHT1;13/MdPHT1;12*, and *MdPHT1;6/MdPHT1;1*.

### Motif analysis of MdPHTs

**Figure 4:**
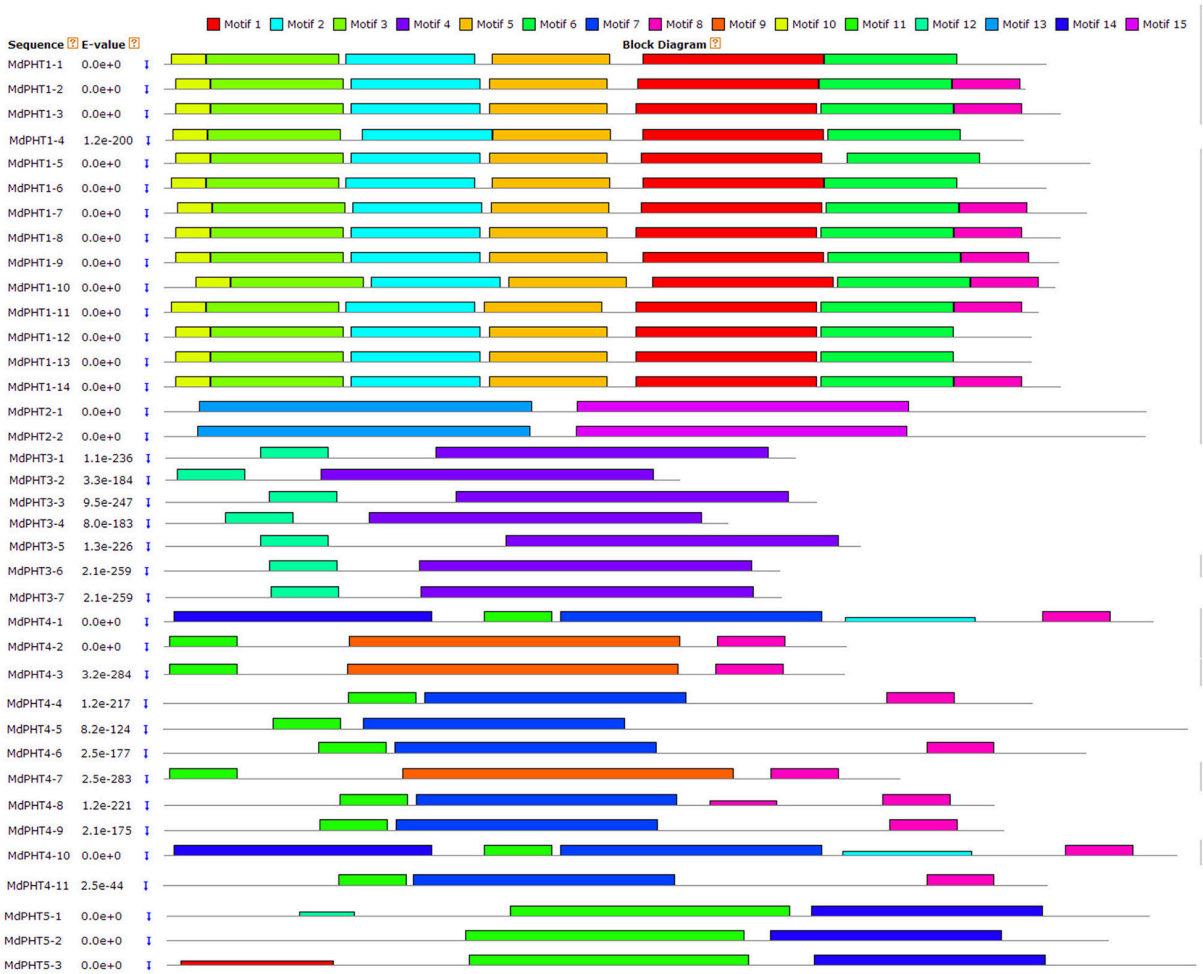
**Protein sequences identified from MdPHT family and aligned with MEME software**.

Based on the protein sequence alignments obtained via MEME software (http://alternate.meme-suite.org/), we identified 15 putative conserved motifs (6–200 aa long) in the MdPHT family (Figure 4). Motifs 1, 2, 3, 5, 10, and 11 were present in all Cluster-I proteins; Motifs 13 and 15 appeared in all Cluster-II proteins; and Motifs 4 and 12 were in all Cluster-III proteins. For Cluster IV, MdPHT4;2, MdPHT4;3, and MdPHT4;7 contained Motifs 6, 8, and 9; MdPHT4;1 and MdPHT4;10 had Motifs 2, 8, and 11; while the others contained Motifs 6 and 7. All Cluster-V proteins featured Motifs 11 and 14.

### Expression profiles of apple PHT genes in different tissues

**Figure 5:**
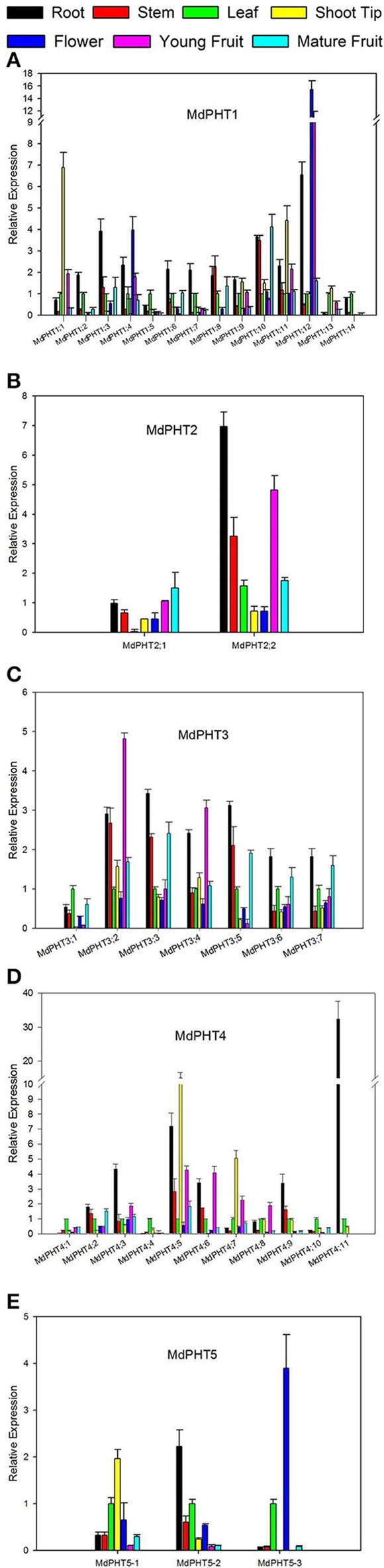
**Quantitative real-time PCR analysis of selected apple MdPHT1 genes (A)**, MdPHT2 genes **(B)**, MdPHT3 genes **(C)**, MdPHT4 genes **(D)**, MdPHT5 genes **(E)** expressed in roots, stems, leaves, shoot tips, flowers, young fruits and mature fruits from *Malus hupehensis* var. *pingyiensis*. Data were normalized to level of apple *Actin* expression. Value for each sample is mean of 3 replicates. Vertical bars indicate standard deviation.

The qRT-PCR analysis revealed ubiquitous expression for all 37 *MdPHT*s in the apple roots, stems, leaves, shoot tips, flowers, young fruits and mature fruits. For Cluster-I members (Figure 5A), expression for *MdPHT1;12* was strongest in roots and flowers, and its transcript levels were approximately three- to 5-fold higher than those of other *MdPHT1* genes. Expression was strongest for *MdPHT1;3* in the roots and for *MdPHT1;5* and *MdPHT1;13* in the leaves. The expression of *MdPHT1;1* and *MdPHT1;11* was 7- to 5-flod than other *MdPHT1s* in shoot tips. Most of *MdPHT1s* showed downtrend in fruit ripening, especial *MdPHT1;10* was expressed highly in young fruits than mature fruits. Genes in Cluster II (Figure 5B) were expressed in all tissue types, but *MdPHT2;2* was most highly expressed in the roots, stems and young fruits. Although genes in Cluster III (Figure 5C) were expressed in almost all tissues, expression was particularly high for *MdPHT3;2* and *MdPHT3;3* in the roots, stems and young fruits. For Cluster IV (Figure 5D), *MdPHT4;1, MdPHT4;4*, and *MdPHT4;10* were expressed only in the leaves, while transcripts of *MdPHT4;11* were detected in the leaves and the roots. Four other genes – *MdPHT4;3, MdPHT4;5, MdPHT4;6*, and *MdPHT4;9*—showed the greatest expression in the roots. The expression of *MdPHT4;5* and *MdPHT4;7* was highly in the shoot tips. Within Cluster V (Figure 5E), *MdPHT5;1* and *MdPHT5;2* were most strongly expressed in the leaves and roots, respectively, while expression of *MdPHT5;3* was highest in the flowers.

### Effects of stress treatment on MdPHT expression

**Figure 6:**
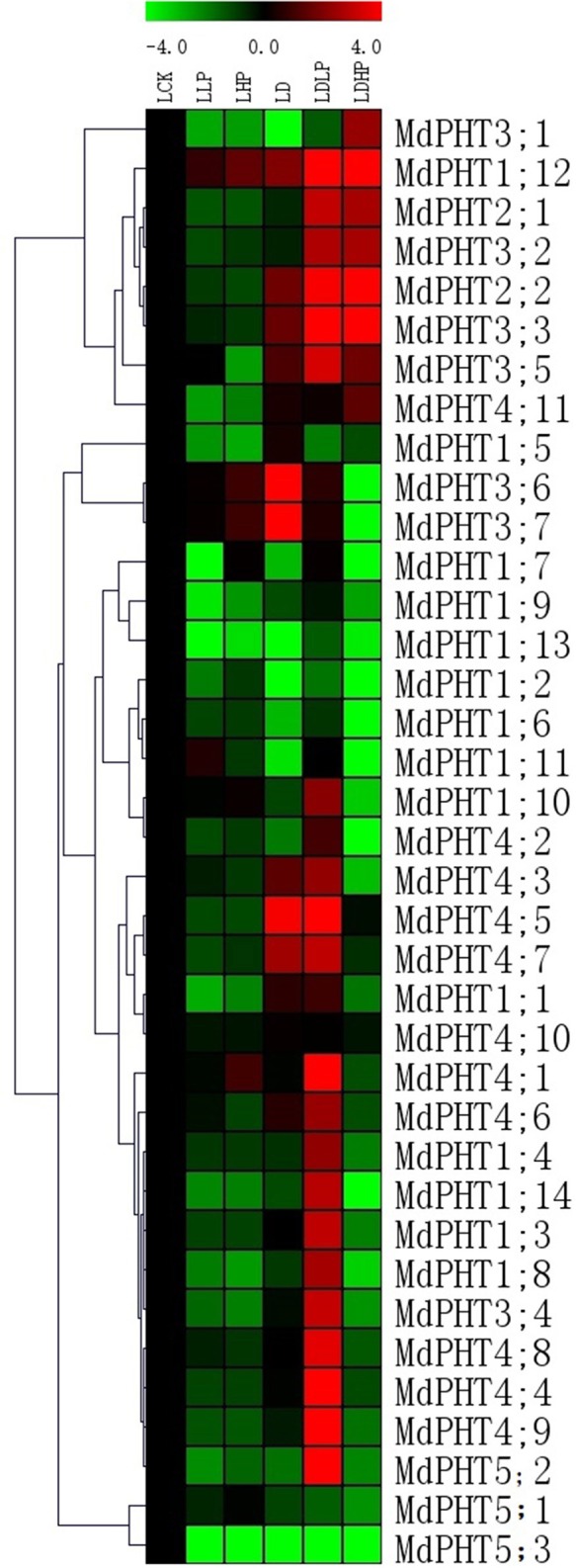
**The expression profiles of *PHT* genes in leaves of *Malus hupehensis* var. *pingyiensis***. Heatmap shows PHT gene expression across after 15 d of phosphorus treatment, samples were taken from leaves exposed to low-P (LLP), high-P (LHP), drought (LD), drought with low-P (LDLP), or drought with high-P (LDHP) conditions. All transcript levels were normalized to their respective corresponding levels in non-stressed control (LCK).

After plants were exposed to 15 d of low-P conditions, only two genes (*MdPHT1;11* and *MdPHT1;12*) were up-regulated in the leaves (Figure 6). The expression of other genes either remained stable or decreased. Under high-P conditions, expression of *MdPHT1;12* was increased in the leaves while that of *MdPHT3;6, MdPHT3;7*, and *MdPHT4;1* was up-regulated in those samples. Expression of *MdPHT1;7, MdPHT1;10, MdPHT4;10*, and *MdPHT5;1* was not significantly changed by such treatment while all other genes were down-regulated. Under drought stress, expression in the leaves increased for *MdPHT3;6, MdPHT3;7, MdPHT4;5, MdPHT1;12* and *MdPHT4;7* when compared with the control. In response to the combined stress of low-P and drought, *MdPHT5;2* and *MdPHT3;3* were significantly induced in the leaves. Under the double stress of high-P and drought, *MdPHT1;12, MdPHT2;2*, and *MdPHT3;1-3;3* were obviously up-regulated. Other genes showed either stable or only slightly decreased expression when plants were exposed to that combined treatment.

**Figure 7:**
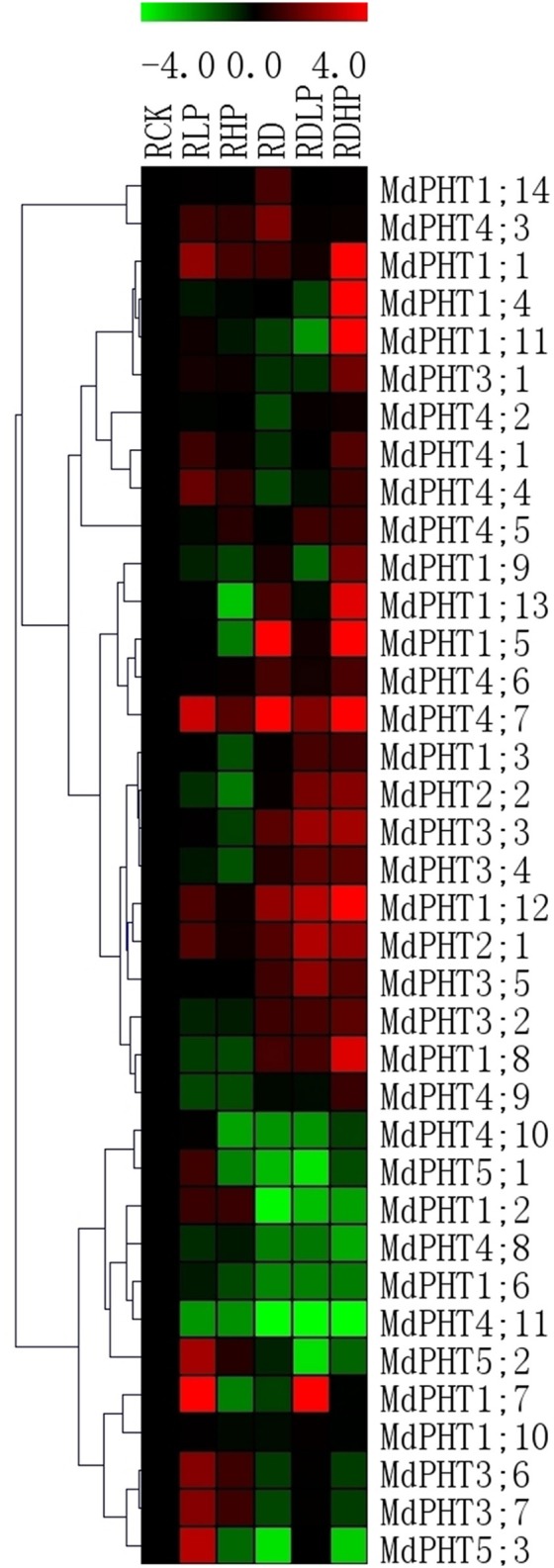
**The expression profiles of *PHT* genes in roots of *Malus hupehensis* var. *pingyiensis***. Heatmap shows PHT gene expression across after 15 d of phosphorus treatment, samples were taken from roots exposed to low-P (RLP), high-P (RHP), drought (RD), drought with low-P (RDLP), or drought with high-P (RDHP) conditions. All transcript levels were normalized to their respective corresponding levels in non-stressed control (RCK).

In the roots, P-starvation treatment led to 14 *MdPHT* genes were highly expressed in the roots of *M. hupehensis*, suggesting that most *MdPHT* genes might be involved in Pi uptake. Only *eight* genes were down-regulated (Figure 7). When excessive P was supplied, expression of *MdPHT4;7, MdPHT1;1, MdPHT1;2, MdPHT3;6, MdPHT3;7*, and *MdPHT4;3-4;5* were induced over the control. Expression of other genes was unchanged or declined after 15 d of high Pi treatment. Under drought stress, the expression of 15 *MdPHT* genes was up-regulated in apple. Expression of *MdPHT1;7* was obviously up-regulated by the combined drought and starvation treatment. All of the *MdPHT2s* and most of the *MdPHT3* members were also induced under such conditions. When drought and high-P stresses were applied together, expression of only *MdPHT1;8, MdPHT1;10, MdPHT1;14, MdPHT4;2*, and *MdPHT4;3* remained stable while that of most other genes was up-regulated. Quantitative real-time PCR analysis of selected apple PHT genes expressed in leaves and roots under different stress treatments showed in Images 1, 2.

## Discussion

Phosphate is one of the most important nutrients that significantly affect plant growth and metabolism (Raghothama, 1999). Therefore, examining the relationship between PHT proteins and Pi uptake/translocation enables us to understand how plants adapt to different environments. Our research was the first to focus on such transporters in apple, and involved a genome-wide survey and characterization of those genes so that potential candidate PHTs can be utilized in future functional analyses.

### PHT genes in apple

We identified 37 members of the apple PHT family based on sequence alignments with the *Arabidopsis* PHT family. These MdPHTs can be classified into five clusters. A BLAST of the NCBI database indicated that the PHT1 family comprises more than 100 proteins. They include 13 *PHT1* genes in the genome of haploid rice (Paszkowski et al., 2002); plus eight in *Hordeum vulgare* (Rae et al., 2003); five each in *Zea mays* (Nagy et al., 2002), *Lycopersicon esculentum*, and *Solanum tuberosum* (Nagy et al., 2005); and four in *Nicotiana tabacum* (Kai et al., 2002).

The MdPHT1 family is larger in apple (14 genes) than in other plants. These PHT1 members exhibit high sequence similarity to each other and contain 12 putative transmembrane segments (Raghothama, 1999; Smith, 2001). In apple, 12 TMSs are conjectural in eight MdPHT1 proteins, while 13 are predicated in six MdPHT1 proteins. The MdPHT1 proteins have 517 to 554 amino acids, making them similar in size to AtPHT1 proteins in *Arabidopsis* (Saier, 2000).

One PHT2 family gene has been identified in *Arabidopsis* (Daram et al., 1999) while two (*MdPHT2;1* and *MdPHT2;2*) occur in apple. The latter two genes are located on different chromosomes (chr13 and chr16), and their encoded proteins are shorter than the AtPHT2;1 protein, i.e., 59 vs. 61 kDa. The 12 TMSs in Cluster II of apple compare with the 11 each in PHT2;1 of *Arabidopsis* and PHT2 of rice (Liu et al., 2011).

*Arabidopsis* has at least three *AtPHT3* genes (Rausch and Bucher, 2002), while apple has seven in that family. The number of TMSs also varies among species, with rice having four to seven and *Arabidopsis* having seven (Liu et al., 2011). We found that apple has seven to eight such transmembrane-domain structures.

Apple contains 11 *MdPHT4* genes while *Arabidopsis* has six (Guo et al., 2008). Rice has 11 to 12 TMSs in that family while *Arabidopsis* and apple each have 12 (Liu et al., 2011). The three MdPHT5 family genes in apple (12 TMSs) show similarities to the PHT5 protein in *Arabidopsis* (Liu et al., 2016). Because most of these MdPHTs (Table 1) are the same length as those members in *Arabidopsis* and rice (Liu et al., 2011), we conclude that proteins within that family are highly conserved among species.

Among the 37 coding sequences for MdPHT proteins, 18 have no introns while the others are disrupted by 1 to 15 introns each. When compared with the gene structures for *Arabidopsis* and rice PHTs (Liu et al., 2011), MdPHT1s have the same number of exons (1-2) as AtPHT1s and OsPHT1s, while MdPHT2s have three exons. All of the MdPHT3s have one exon fewer than either AtPHT3s or OsPHT3s while both MdPHT4s and MdPHT5s have more exons than the other two species. This difference suggests that the gene structure in apple is more divergent than that in *Arabidopsis* or rice.

### Transcript profiles of MdPHT genes in different tissues

Most *AtPHT1* genes are strongly expressed in the roots but are also detected in leaves and pollen (Karthikeyan et al., 2002; Mudge et al., 2002; Schünmann et al., 2004). Eight of the nine PHT1 family genes in *Arabidopsis* are expressed in the roots (Bucher et al., 2001; Mudge et al., 2002). By comparison, eight *PHT1* genes in tomato (Chen et al., 2014) and 10 of the 13 *PHT1* genes in rice are expressed in the roots (Paszkowski et al., 2002). We also detected *MdPHT1;1, MdPHT1;2, MdPHT1;7, MdPHT1;13*, and *MdPHT1;14* transcripts in both leaves and roots. Expression of *MdPHT1;12* is strong in the roots, flowers and young fruits. Almost all of the proteins in this family are predicted to be localized to the plasma membrane and expressed in the roots, suggesting their role in Pi uptake at the root–soil interface.

The PHT2 family may be low-affinity Pi transporters (Daram et al., 1999). PHT2;1 is preferentially expressed in the shoots, especially in rosette leaves (Ferro et al., 2002; Versaw and Harrison, 2002). We cloned two *MdPHT2* genes and found that both are expressed in roots, stems, leaves, shoot tips, flowers, young fruits and mature fruits. As also noted for AtPHT2;1, a chloroplast phosphate transporter (Ferro et al., 2002; Versaw and Harrison, 2002), proteins of MdPHT2 are localized to that organelle.

Plant PHT3s are highly conserved within the mitochondrial transporter family (Laloi, 1999). In apple, MdPHT3 proteins are also predicted to occur on the mitochondria. Their strong expression in the roots, stems, leaves, shoot tips, flowers, young fruits and mature fruits are evidence of their great importance for plant growth.

The six members of the *PHT4* gene family in *Arabidopsis* are well-characterized, putative intracellular PHTs (Guo et al., 2008). Such genes are generally expressed in roots and leaves. In apple, *MdPHT4;1, MdPHT4;4*, and *MdPHT4;10* are expressed predominantly in the leaves while *MdPHT4;3, MdPHT4;5, MdPHT4;6, MdPHT4;9*, and *MdPHT4;11* are expressed predominantly in the roots. In particular, expression is approximately 30-fold greater for *MdPHT4;11* than for other family members.

PHT5 is considered a vacuolar phosphate transporter. Whereas *AtPHT5;1* from *Arabidopsis* is expressed in most tissue types, *AtPHT5;2* expression is confined to the guard cells, vascular tissue, and pollen (Liu et al., 2016). In apple, *MdPHT5s* genes are expressed less in young fruits and mature fruits. The expression of *MdPHT5;2* is high in the roots while that of *MdPHT5;3* is detected mainly in the stems and flowers. Because MdPHT5 proteins are localized to the vacuoles, this implies that they have possible functions in Pi storage within different tissues.

### Expression of PHT genes in response to different phosphate levels and drought in apple

Transcription of *MdPHT* genes varies in response to changes in phosphate levels. We found that, upon Pi-starvation, expression of *MdPHT1;1, MdPHT1;2, MdPHT1;7, MdPHT1;12, MdPHT3;6, MdPHT3;7, MdPHT4;7*, and *MdPHT5*s is induced in the roots. Most PHT1 family genes expressed in *Arabidopsis* roots are up-regulated in phosphate-deprived plants (Mudge et al., 2002). We also found that starvation conditions led to increases in expression, *MdPHT1;1* and *MdPHT1;7* increasing greatly over the control. *MdPHT1;7* in apple are homologous to *AtPHT1;4*, which have been shown to be strongly up-regulated during Pi starvation (Shin et al., 2004). Indicating that they are maybe high-affinity phosphate transports proteins that operate at low Pi concentration by moving Pi from the external medium to the cytosol. Three *MdPHT5* genes, like *AtPHT5s* (Liu et al., 2016), all transporters in the vacuoles, are markedly up-regulated in the roots under low-Pi conditions, supporting their roles in storing or removing phosphate. Although most *MdPHT* genes are up-regulated in the roots, two of them – *MdPHT1;11* and *MdPHT1;12*—are also induced in the leaves while expression of the others remains stable or decreases in those tissues. Thus, genes expressed in the roots may be more sensitive to a limited supply of P in the soil.

When plants are exposed to excess Pi, *MdPHT3;6, MdPHT3;7, MdPHT4;5*, and *MdPHT4;7* are up-regulated in the roots, suggesting that their proteins are low-affinity transporters. This might also be true for other PHT4 family members, based on the relatively high levels of Pi reported in the cytosol and other subcellular compartments (Mimura, 1999). It is very interesting that *MdPHT3;6* and *MdPHT3;7* showed up-regulated in roots and leaves under high Pi condition. AtPHT3 was a mitochondrial phosphate transporter (Jia et al., 2015). Under adversity stress, faster respiration rate and more reactive oxygen species (ROS) were produced in the plants, *MdPHT3;7* and *MdPHT3;8*, predicted located in mitochondrial, were induced to transport more Pi to utilize for the conversion of ADP to ATP.

In response to drought treatment, expression of *MdPHT1.5, MdPHT1;7*, and *MdPHT4;7* is markedly up-regulated in stressed apple roots, while *MdPHT3;7*, and *MdPHT4;3* are obviously induced in the leaves. The expression levels of *PtPHT1;2*, and *PtPHO9* were up-regulated under drought condition in poplar (Zhang et al., 2016). This suggests that these genes are positively regulated by drought stress. In contrast, experiments involving low-P conditions, alone or in combination with drought, showed that expression of *MdPHT1;7* are extremely response. When only drought is induced, expression of that gene remains stable. It just induced by low Pi treatment, indicating that the up regulation of *MdPHT1;7* may contribute to a low level of Pi tolerance of apple plants. Regardless of Pi status, expression of *MdPHT1;8, MdPHT1;12, MdPHT3;2-3;5*, and *MdPHT4;7* in the roots, and *MdPHT2;2, MdPHT3;3*, and *MdPHT3;5* in the leaves, is up-regulated under drought conditions, implying that these genes are not influenced by phosphate levels. However, drought-related changes in expression of *MdPHT1;1, MdPHT1;5, MdPHT1;7, MdPHT1;11, MdPHT4;7*, and other genes are largely dependent upon the level of phosphate. These results suggest therefore that, under drought conditions, Pi uptake in plants is likely associated with alterations in *PHT* expression, and may reflect the degree of drought tolerance in those stressed plants.

Under the drought and low phosphorus condition, the expression of 23 *MdPHT* genes is up-regulation in the leaves, 12 genes are induced in the roots, suggesting that the plant of aerial parts are influenced greatly than the underground parts, the aboveground parts of plant are not only to fight drought, but also to strengthen reusing the phosphorus. Relative to the single stress, *MdPHT* genes response positively under the dual stresses, the mechanism of the deeper reason remains to be further research.

The expression of *MdPHT4;10* is concentrate upon leaves and it has nothing to do with the stresses, the subcellular location predicted of *MdPHT4;10* is on chloroplast, so we guess MdPHT4;10 like AtPHT4;4 may be a chloroplast-localized ascorbate transporter (Miyaji et al., 2015).

Numerous PHT family genes have been described for rice and *Arabidopsis* but not for apple. Our study is the first to screen and analyze that family in apple and investigate their expression in different tissues, as well as under drought stress or limited/excessive Pi. Additional research is necessary if we are to understand the biological functions and regulatory mechanisms by which plants adapt when exposed to adverse growing environments.

## Conclusions

We identified and characterized 37 *MdPHT* genes and organized them into five clusters based on their distinct exon/intron structures, phylogeny, and distribution of conserved motifs. Our qRT-PCR results demonstrated that these genes are induced by both Pi-starvation and excessive phosphate levels, as well as under drought stress. Therefore, they are good candidates for further analysis of their activities and functions. Although the roles for such MdPHTs are not yet fully known, our findings provide evidence that they participate in plant adaptations to stress conditions. If they can then be exploited to improve the tolerance of apple to Pi-starvation stress, then our study will also serve as a framework for future functional studies of the PHT family in that crop.

## Author contributions

FM and ML conceived and designed the experiments. TS and LY performed the experiments. TS, YS, and ML analyzed the data. TS, ML, and FM wrote the paper.

### Conflict of interest statement

The authors declare that the research was conducted in the absence of any commercial or financial relationships that could be construed as a potential conflict of interest.

## Supplementary material

Image 1 **Expression patterns for selected apple *PHT* genes in leaves of *Malus hupehensis* var. *pingyiensis***. After 15 d of phosphorus treatment, samples were taken from leaves exposed to low-P (LLP), high-P (LHP), drought (LD), drought with low-P (LDLP), or drought with high-P (LDHP) conditions. All transcript levels were normalized to their respective corresponding levels in non-stressed control (LCK). Apple *Actin* served as reference gene. Mean values were obtained from 3 replicates. Vertical bars indicate standard deviation.

Image 2 **Expression patterns for selected apple *PHT* genes in roots of *Malus hupehensis* var. *pingyiensis***. After 15 d of phosphorus treatment, samples were taken from roots exposed to low-P (RLP), high-P (RHP), drought (RD), drought with low-P (RDLP), or drought with high-P (RDHP) conditions. All transcript levels were normalized to their respective corresponding levels in non-stressed control (RCK). Apple *Actin* served as reference gene. Mean values were obtained from 3 replicates. Vertical bars indicate standard deviation.

Table S1 **Primers used for cloning of *MdPHT* genes and quantitative real-time PCR analyses**.

## Abbreviations

EST: expressed sequence tag
PHT: phosphate transporter
qRT-PCR: quantitative real-time polymerase chain reaction
DAB: days after blooming.
