# Supplementary figures and images for: Comprehensive Genomic Identification and Expression Analysis of the Phosphate Transporter (PHT) Gene Family in Apple
Source: Front Plant Sci. 2017 Mar 30;8:426. doi: 10.3389/fpls.2017.00426 (PMC5371654; doi:10.3389/fpls.2017.00426)

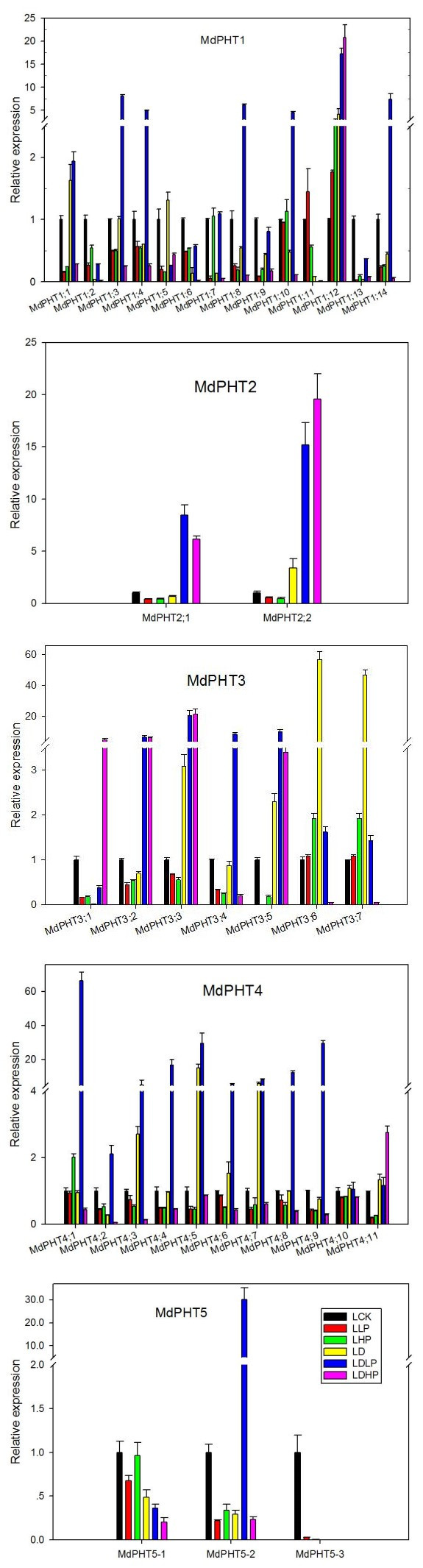

Supplement: Image 1 — Expression patterns for selected apple PHT genes in leaves of Malus hupehensis var. pingyiensis. After 15 d of phosphorus treatment, samples were taken from leaves exposed to low-P (LLP), high-P (LHP), drought (LD), drought with low-P (LDLP), or drought with high-P (LDHP) conditions. All transcript levels were normalized to their respective corresponding levels in non-stressed control (LCK). Apple Actin served as reference gene. Mean values were obtained from 3 replicates. Vertical bars indicate standard deviation. [file Image1.JPEG]

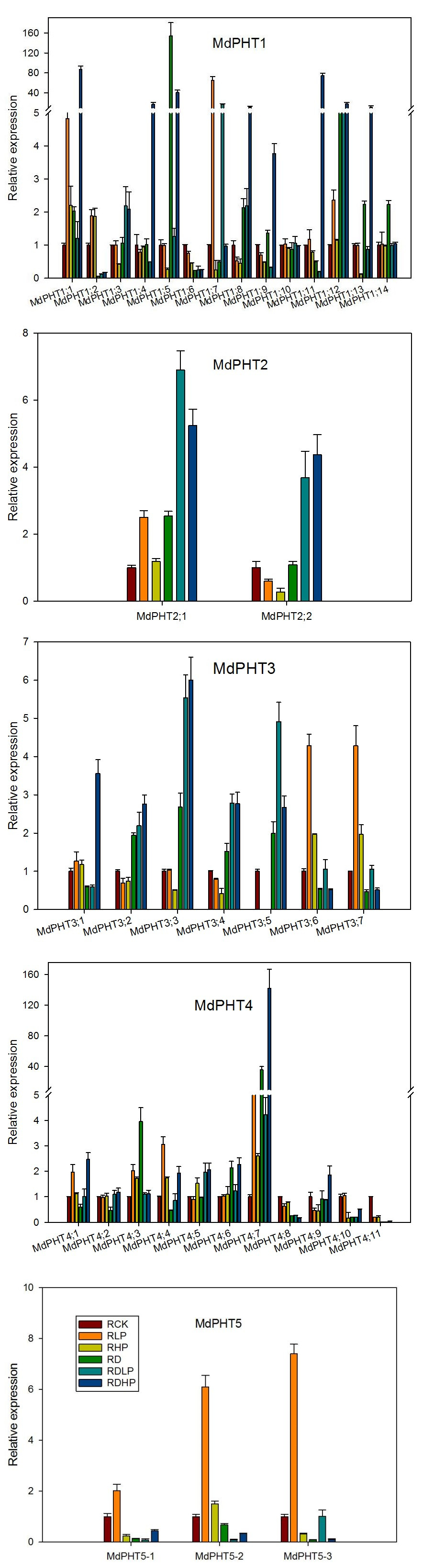

Supplement: Image 2 — Expression patterns for selected apple PHT genes in roots of Malus hupehensis var. pingyiensis. After 15 d of phosphorus treatment, samples were taken from roots exposed to low-P (RLP), high-P (RHP), drought (RD), drought with low-P (RDLP), or drought with high-P (RDHP) conditions. All transcript levels were normalized to their respective corresponding levels in non-stressed control (RCK). Apple Actin served as reference gene. Mean values were obtained from 3 replicates. Vertical bars indicate standard deviation. [file Image2.JPEG]
