# Supplementary material for: Comprehensive Genomic Identification and Expression Analysis of the Phosphate Transporter (PHT) Gene Family in Apple
Source: Front Plant Sci. 2017 Mar 30;8:426. doi: 10.3389/fpls.2017.00426 (PMC5371654; doi:10.3389/fpls.2017.00426)
Supplement: Table S1 — Primers used for cloning of MdPHT genes and quantitative real-time PCR analyses. [file Table1.DOCX]

| Gene name | Primers for cloning (5’–3’ ) | | Primers for qRT-PCR (5’–3’) |
| --- | --- | --- | --- |
| MdPHT1;1 | 5' ATGGCCATAGCCGTGCTGC 3'  5' CTAGTACGGCGTGGCTGTTGTTG 3' | 5' ACTCAAAAGCCCGAGGACGT 3'  5' GAACCAGTAACCAGGGAAGG 3' | |
| MdPHT1;2 |  | 5' CCGTAAATGGTGTGGCGTT 3'  5' CATGATTGTGGCAGAAAGAGG 3' | |
| MdPHT1;3 |  | 5' TGGTGACAAGATGGGAAGGA 3'  5' ATTGTGGCAGAAAGAGGGTAG 3' | |
| MdPHT1;4 |  | 5' TCACCGACGCATACGATC 3'  5' CAAGCCAGCCGAAGACC 3' | |
| MdPHT1;5 |  | 5' CCGCCTTTACTACTACGACCCT 3'  5' TTCCGAACAGAGCAACCC 3' | |
| MdPHT1;6 |  | 5' CTTCTTCAGGTTTTGGTTAGGG 3'  5' CGAGTCATTTTGTTGGCGTAT 3' | |
| MdPHT1;7 |  | 5' CTTCCCAGCAAGGTTGAGGT 3'  5' TGCCGAAGAAGTTGATGACG 3' | |
| MdPHT1;8 |  | 5' TGGTGACAAGATGGGAAGGA 3'  5' AAACGAGGCCGCAGAGATGA 3' | |
| MdPHT1;9 | 5' ATGGCTAGAGAGCAATTGGGAGTG 3'  5' TCAAACTGGAACGGTTCTAGCAGAA 3' | 5' CCGACCTAACTCACCCAAAAC 3'  5' CTGACGCAACGGAGCATAAA 3' | |
| MdPHT1;10 |  | 5' TCCTTACCACCACTGGATTAGA 3'  5' CCACTGGCACAACAAACG 3' | |
| MdPHT1;11 |  | 5' GGGTGGTGGCTATTGTGGT 3'  5' ACGCCTTGCTTTGATCCTT 3' | |
| MdPHT1;12 |  | 5' AGTTTGCCTCATAATGTTTCG 3'  5' CATAAGGACCATGCCGTAGA 3' | |
| MdPHT1;13 |  | 5' TGGACACTAAAAGAAAACCGAG 3'  5' GTACTGAAATCCGAAAGCAC 3' | |
| MdPHT1;14 |  | 5' GCGGACTATCTGTGGAGGAT 3'  5' CAACAAGGGCGGTGTAACG 3' | |
| MdPHT2;1 | 5' ATGATTCCTTCTTACTGCCTATCTT 3'  5' TCATAAAATATAAGACAAAAGCTTGGT 3' | 5' CCTCGGTGGTTCTATTTGCTTC 3'  5' CCTCACGGTTTCTGCTCTAACTCTA3' | |
| MdPHT2;2 | 5' ATGACTCCTTCCTACTGCTTATCTT 3'  5' TCACAAAATATAAGATAAAAGCTTGGT 3' | 5' GAATGATGTCTCCAACGCAATA 3'  5' GAATAACAATCTCCGCTCCACT 3' | |
| MdPHT3;1 | 5' ATGGCAGATCCACTCAGCAACTCTC 3'  5' TCAAGACTTCGCAACCTCGATGGCG 3' | 5' CTGGGCTGGTTACAGTGTTCAGG 3'  5' GACGGCTTCCATAGGACAGAGGG 3' | |
| MdPHT3;2 |  | 5' CCATCTCGCAATCACGCCACT 3'  5' TGCAGCCACCCTGAACACCAT 3' | |
| MdPHT3;3 |  | 5' CCCTAGACCTAAGAGCGAGTGC 3'  5' CAAGATTGTCAGCAGGATGAGATA 3' | |
| MdPHT3;4 |  | 5' ACACGACACGGGTTGAAGGAA 3'  5' GTAGCGCCAGCACTGAGCATT 3' | |
| MdPHT3;5 |  | 5' TCATTGCCGATGTTGCCCTCTGT 3'  5' AAGGCCATCAGCCAAGCCTCTGG 3' | |
| MdPHT3;6 |  | 5' CTCAGATAAGCAGTCGCGTCAGG 3'  5' AAGTACAGGCAGCGTAGAAGGAA 3' | |
| MdPHT3;7 |  | 5' TTTGGTTTGTATCGGAGACATTTGT 3'  5' TGTGATGCTTTTGTACTTTGCAGGA 3' | |
| MdPHT4;1 |  | 5' AGTTTGCCTCATAATGTTTCG 3'  5' CATAAGGACCATGCCGTAGA 3' | |
| MdPHT4;2 |  | 5' GTTGCCAATCAATGGAAGTCA 3'  5' GGCCCAATTCAAAGTATGTCG 3' | |
| MdPHT4;3 | 5' ATGAGGACGATGAAATTTCCAAAG 3'  5' TCAGTCAAAAATTCTCTCCCCC 3' | 5' TTATATTCCCCCTCAATCCACACAG 3'  5' AAGCAAAGACCACATGGCACCTAGT 3' | |
| MdPHT4;4 | 5' ATGAATGCCAGAGCACTTCTTTAC 3'  5' CTAATCTATGACCTTTTCGCCAG 3' | 5' ATTTGGAGTCATTTGGTGGTC 3'  5' TTATTCATGGCAGGCATAGC 3' | |
| MdPHT4;5 |  | 5' CCATCGTCCCTCTTGCTGCTAAAA 3'  5' CACACCCCATGCCATCACTCTCTT 3' | |
| MdPHT4;6 | 5' ATGGCCTCCGCAGTCCG 3'  5' TTATTCATCGATACAACGGATGAC 3' | 5' GGGTTTCTTCCAACGACGCTCAG 3'  5' ACATCACGACACGGTCCGCATT 3' | |
| MdPHT4;7 | 5' ATGGGGACGATGAAATTTCCAAAG 3'  5' GCAGGAGGTATGCATGGAAAAAAC 3' | 5' CAGTTTGGGCAATCGTGGTTAA 3'  5' AAAGTATGTCGGCAGCCAGTTC 3' | |
| MdPHT4;8 | 5' ATGAACCCCAGAGCGCTTC 3'  5' CTAATCTATGATCTTTTCGCCAGTT 3' | 5' ACTTGTTGTTCGTGCCTTCA 3'  5' TTCCACTGTAAACCAGAGCC 3' | |
| MdPHT4;9 |  | 5' TGGCGGTCATGGGATATGTT 3'  5' TTCAGCCCGACTGCTAAAGT 3' | |
| MdPHT4;10 | 5' ATGGCGATCGGCGGTTTG 3'  5' CTAGTCAATAATTTTCTCCCCCGT 3' | 5' ACCTTGGTTCTGTCACTGGG 3'  5' CTTTTGGTGTGCTATATGCTTTA 3' | |
| MdPHT4;11 |  | 5' CGTTCCCTCCGAAGCATAAT 3'  5' TTCACCCTCAGAATCCGACCC 3' | |
| MdPHT5;1 | 5' ATGGTCGCCTTCGGGAAGAAGCTGC 3'  5' ATGGTCGCCTTCGGGAAGAAGCTGC 3' | 5' TCTTGCGTGGCTCATCTATCTGTT 3'  5' TTCTTCGGCATCCTCACCATCTGT 3 | |
| MdPHT5;2 | 5' ATGAAGAAAAAAGTAAGACACTATT 3'  5' TCAATAGAGAGAGTTGTATGTGTAG 3' | 5' ATTAGTGCTGAAGACAAGAGAGAAG3'  5' ACAGAGGGTGTAAGTAGTTGATATG 3' | |
| MdPHT5;3 | 5' ATGGTTGCCTTTGGGAAAAAGCTGA 3'  5' TCAATAGAGAGAGTTGTACGTGT 3' | 5' CACCCAAGAACTCAAATTCCCATT 3'  5' GAAGCTTTAGAAGATCACTCCCCA 3' | |
| Action |  | 5' AACAATGCTAGGGAACACGGCTCT 3'  5' ACAGGAAGTAGAAGATGGCGGACA 3' | |
